# Improving our patients' experience: ideas for change

**Published:** 2012

**Authors:** Sally Crook, Boateng Wiafe

**Affiliations:** Programme manager: Seeing is Believing and Consulting Editor for this issue.; Regional Director for Africa, Operation Eyesight Universal

**Figure:**
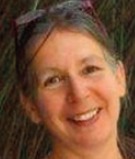
Sally Crook

**Figure:**
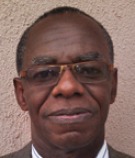
Boateng Wiafe

In addition to keeping an eye on the quality of our clinical services, making small changes in how we deliver services can have a big impact on how patients feel about their experience – which will in turn motivate them and others to come for the eye care they need. Here are a few things to you may be able to try.

## Look after your staff

Patients want us to listen to them. They want to be treated with dignity and respect. If staff are well trained and confident in what they do, they will be less stressed, more relaxed, and will find it easier to listen and talk to patients. Staff who feel valued and respected by their managers are also more likely to treat their patients with respect. In addition, consider giving staff specific training in how to be welcoming, caring, and supportive. Give recognition to staff members who do this well!

## Be informed about other services

Find out what other health and support services are available in your area, for example low vision, rehabilitation, inclusive education, as well as clinics such as diabetes, neglected tropical diseases, neonatal units, and so on. Visit them and invite them to visit your service. Ask for leaflets or posters of their services, and ensure you know what their opening times and days are so patients do not have wasted journeys.

## Get involved

Find out what other organisations or groups work with potential users of your eye service. For example, an organisation providing services to pensioners or other older people, a school for the blind, white cane training events, or the national society for the blind (where it exists). Think about how you can support their activities and share information with them.

## Set specific times for clinics

These should be displayed both inside and outside the building, for example using posters or signboards. Give printed information about clinic times and dates to other health and related staff in your area, for example, primary health care workers, mother and child workers, birth attendants, pharmacists, and optometrists. Then the community and patients will know when they should come for nonemergency treatment and how long a wait to expect. This can help you to manage your patients’ visits and eliminate wasted journeys.

## Plan around your patients

Think about your patients. Do they know where to go? Are there long waiting times? Are the receptionists friendly, and do they explain to patients what to expect? Every now and then, take some time to put yourself in your patients’ shoes (see ‘Time to reflect’, opposite page). If you were a patient in your clinic, how would you feel? What would make you feel better?

## Explain costs clearly

All costs for services should be clearly given in advance – whether verbally, on paper, or using posters and signboards.

Include all services covered by insurance schemes or other waivers. Patients then know what to expect and can make informed decisions. This will also make it difficult for anybody to ask patients to pay more than the authorised amount.

**‘All costs for services should be clearly given in advance/**

## Learn from others

Ask for assistance and find ways of sharing skills and experiences with your colleagues, for example ophthalmic nurses or ophthalmic clinical officers at other clinics or hospital in your area. If you don't ask, you will not know how you may be able to help each other. Visit other clinics or hospitals to see how they put patients at the centre of what they do.

## A focus on quality

The World Health Organization's standards for surgery^1^ and other outcomes should be the benchmark used in all eye care teams. This will inspire confidence in patients and their families. Successful outcomes are the best advertisement for your services in particular and eye care services in general.

**Figure:**
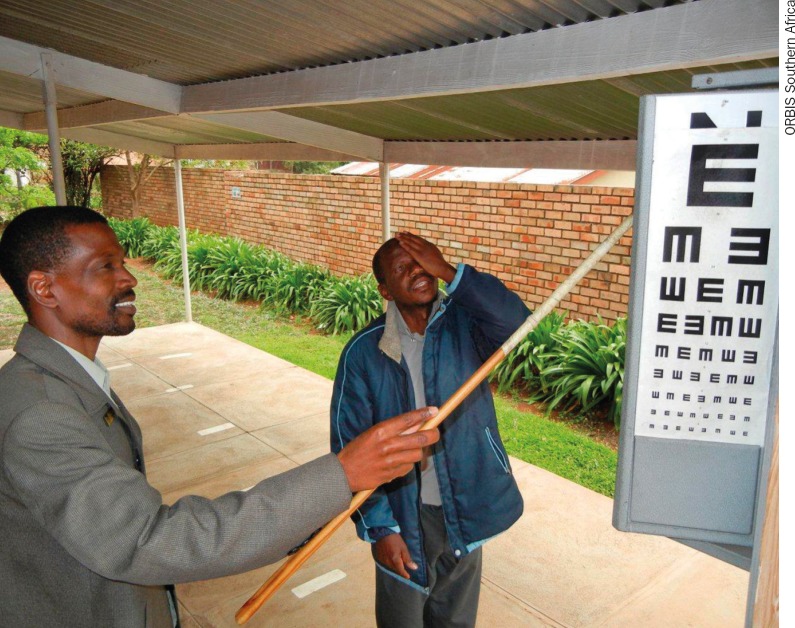
If staff are well trained and treated with respect by their managers, they will enjoy their work and be friendlier to patients. ZAMBIA
